# Advances in Functional and Metabolic Imaging for Early Tumor Treatment Response and Resistance Evaluation: A Review

**DOI:** 10.3390/cancers18050858

**Published:** 2026-03-07

**Authors:** Dengwei Gan, Wenhui Ma, Huan Jie, Cong Huang, Fang Xu

**Affiliations:** 1 Department of Radiology, Chongqing Hygeia Hospital, Chongqing 401331, China; gals_ray@163.com; 2 Department of Radiology, No. 926 Hospital, Joint Logistics Support Force of PLA, Kaiyuan 661699, China; 3 Department of Oncology, No. 926 Hospital, Joint Logistics Support Force of PLA, Kaiyuan 661699, China; 4 Department of Nuclear Medicine, Chongqing Hygeia Hospital, Chongqing 401331, China

**Keywords:** functional imaging, metabolic imaging, tumor treatment, early response, resistance evaluation, positron emission tomography, tumor heterogeneity, immune microenvironment

## Abstract

Early assessment of tumor treatment response and elucidation of resistance mechanisms are critical for optimizing therapeutic strategies and improving patient outcomes. Functional and metabolic imaging technologies, particularly positron emission tomography (PET) combined with specific tracers, enable dynamic monitoring of tumor cell metabolism and microenvironmental changes during the initial phases of therapy. This capability facilitates early prediction of treatment efficacy and investigation into mechanisms underlying drug resistance. This review synthesizes recent advances in the application of functional and metabolic imaging for early tumor treatment response evaluation and resistance assessment. Emphasis is placed on integrating multimodal imaging techniques with molecular biology approaches to comprehensively analyze the relationships among imaging biomarkers, tumor heterogeneity, immune microenvironment, and molecular pathways. The article further explores the clinical translational potential of these imaging modalities while addressing current challenges and limitations. By providing an updated overview of this rapidly evolving field, this review aims to guide future research and clinical application toward more precise and personalized oncology care.

## Simple Summary

Functional and metabolic imaging (e.g., PET, CEST-MRI) is pivotal for early tumor response assessment and deciphering drug resistance, outperforming traditional anatomical imaging. This review synthesizes key technologies (e.g., ^18^F-FDG, hyperpolarized ^13^C-MRI) and their roles in predicting therapeutic efficacy and characterizing tumor heterogeneity. It critically evaluates limitations, clinical validation, and variability of imaging biomarkers, distinguishing between actionable clinical tools and investigational candidates. Addressing real-world barriers (cost, standardization), it outlines future directions to advance imaging-guided precision oncology.

## 1. Introduction

The assessment of treatment efficacy in oncology has traditionally relied on anatomical imaging and clinical symptoms, which often lag behind the dynamic changes occurring within the tumor microenvironment and cellular metabolism. This delay can hinder timely and precise interventions that are crucial for improving patient outcomes. Recent advancements in functional and metabolic imaging technologies have emerged as promising solutions to this challenge. These modalities, which include techniques that measure tumor cell metabolic activity, proliferation status, and immune responses, offer more sensitive indicators of treatment response, particularly in the early evaluation of therapies such as radiotherapy, chemotherapy, and targeted treatments. The integration of novel tracers and the convergence of multi-omics technologies have significantly advanced the understanding of tumor heterogeneity and the mechanisms underlying drug resistance, thus facilitating the development of personalized treatment strategies.

Functional and metabolic imaging techniques provide a unique window into tumor biology by capturing not just the structural changes associated with tumor growth, but also the underlying biochemical processes that drive tumor behavior. For instance, imaging modalities such as positron emission tomography (PET) and magnetic resonance imaging (MRI) can visualize metabolic pathways, allowing clinicians to assess how tumors respond to therapies at a molecular level. This capability is particularly critical when evaluating therapies that may not produce immediate anatomical changes but still lead to significant metabolic alterations indicative of treatment efficacy. As a result, these imaging approaches are increasingly recognized as essential tools in the early assessment of treatment response and the identification of potential resistance mechanisms.

In recent years, the development of innovative tracers has further enhanced the utility of functional and metabolic imaging in oncology. For example, tracers that target specific metabolic pathways or tumor-associated markers can provide insights into the tumor’s biological behavior and its interaction with the surrounding microenvironment. However, the field remains plagued by unresolved controversies, including variable tracer specificity across tumor types, inconsistent clinical validation of novel agents (e.g., ^18^F-VC701), and unclear decision thresholds for imaging-guided treatment adjustments. This specificity not only aids in the accurate characterization of tumors but also helps in monitoring their response to treatment. Moreover, the incorporation of advanced imaging techniques, such as chemical exchange saturation transfer (CEST) MRI and hyperpolarized carbon-13 MRI, has opened new avenues for real-time monitoring of metabolic changes within tumors, thus allowing for more dynamic and responsive treatment planning.

The application of functional and metabolic imaging extends beyond mere assessment of treatment response; it also plays a pivotal role in unraveling the complexities of tumor heterogeneity and resistance mechanisms. A key advantage of imaging-derived tumor heterogeneity over biopsy-based molecular heterogeneity assessment is its ability to capture the spatial and temporal dynamics of tumor subclones in the entire tumor mass, rather than providing a static, localized snapshot from a single biopsy sample that is prone to sampling bias. By elucidating the metabolic profiles of different tumor subtypes and their responses to various therapies, these imaging techniques can inform more tailored and effective treatment strategies. For example, understanding the metabolic adaptations that occur in response to targeted therapies can guide the selection of subsequent treatment options and help mitigate the development of resistance. Furthermore, the integration of imaging data with genomic and proteomic information enhances the ability to predict treatment outcomes and refine therapeutic approaches, ultimately leading to better patient stratification and individualized care. Notably, this integration requires rigorous statistical approaches to mitigate the risks of overfitting and false discovery when correlating high-dimensional imaging features with multi-omics data—challenges that are central to the current limitations of imaging-omics translational research.

As the field of oncology continues to evolve, the integration of functional and metabolic imaging into clinical practice is poised to transform the landscape of cancer treatment. However, widespread adoption is hindered by practical barriers including high equipment and tracer costs, limited accessibility in resource-constrained settings, and workflow incompatibility with routine oncology care—factors that are often overlooked in descriptive reviews but are critical for clinical translation. By providing a more comprehensive understanding of tumor biology and treatment dynamics, these imaging modalities will not only enhance the precision of treatment evaluations but also facilitate the development of innovative therapeutic strategies that are better aligned with the unique characteristics of each patient’s disease. This shift towards personalized medicine, driven by advances in imaging technology, holds great promise for improving patient outcomes and advancing the field of oncology as a whole.

## 2. Overview of Functional and Metabolic Imaging Techniques

### 2.1. Principles of Positron Emission Tomography (PET) Technology

PET imaging leverages positron-emitting radiotracers to quantify biological processes (e.g., glucose metabolism, cellular proliferation) in vivo, with its core clinical value rooted in detecting metabolic alterations prior to anatomical changes—critical for early treatment response assessment in oncology [1]. The most widely used tracer, ^18^F-FDG, mimics glucose and preferentially accumulates in metabolically active tumor cells, with signal detected via gamma-ray annihilation and reconstructed into quantitative metrics (SUVmax, MTV, TLG) that enable objective treatment response evaluation [2]. Hybrid modalities (PET/CT, PET/MRI) integrate functional metabolic data with high-resolution anatomical localization, addressing the low anatomical resolution limitation of standalone PET and improving the differentiation of benign vs. malignant lesions, particularly in heterogeneous tumors [3].

The quantitative capacity of PET enables early response monitoring as early as 2 weeks post-therapy initiation—far sooner than conventional anatomical imaging, which often requires 6–8 weeks to detect volume changes [4]. However, tracer selection directly impacts diagnostic performance: while ^18^F-FDG is broadly applicable across malignancies, it exhibits poor specificity in tumors with low metabolic activity (e.g., indolent prostate cancer, well-differentiated neuroendocrine tumors) and can yield false positives due to physiological uptake in inflamed tissues [5]. This limitation has spurred the development of targeted tracers (e.g., ^18^F-FLT for proliferation, ^68^Ga-FAPI for stromal remodeling), though their clinical validation remains incomplete for most tumor types [6].

A persistent unresolved challenge is inter-scanner and inter-protocol variability of core metrics (SUVmax, MTV, TLG), with reported differences of 20–40% across institutions driven by scanner calibration, reconstruction algorithms, and patient preparation protocols [7]. While international guidelines (e.g., EANM, RSNA) have proposed harmonization strategies (standardized phantom calibration, uniform acquisition parameters), widespread implementation remains limited in community hospitals, hindering cross-center comparisons and standardized treatment decision-making [7]. This variability, coupled with the lack of universal response thresholds across tumor types, underscores a critical controversy in the field: balancing the utility of PET as a quantitative tool with the practical barriers to standardization.

Hybrid PET/MRI offers unique advantages for central nervous system and abdominal tumors, including superior soft tissue contrast and absence of ionizing radiation, but its adoption is constrained by higher cost, longer scan durations (90+ min), and limited availability in resource-constrained settings [3]. In contrast, PET/CT remains the most widely accessible hybrid modality, though its utility is compromised by limited soft tissue contrast and radiation exposure—particularly relevant for long-term monitoring in pediatric or young adult patients [3]. These trade-offs highlight the need for personalized modality selection based on tumor type, patient characteristics, and clinical context, rather than a one-size-fits-all approach.

**Figure 1:**
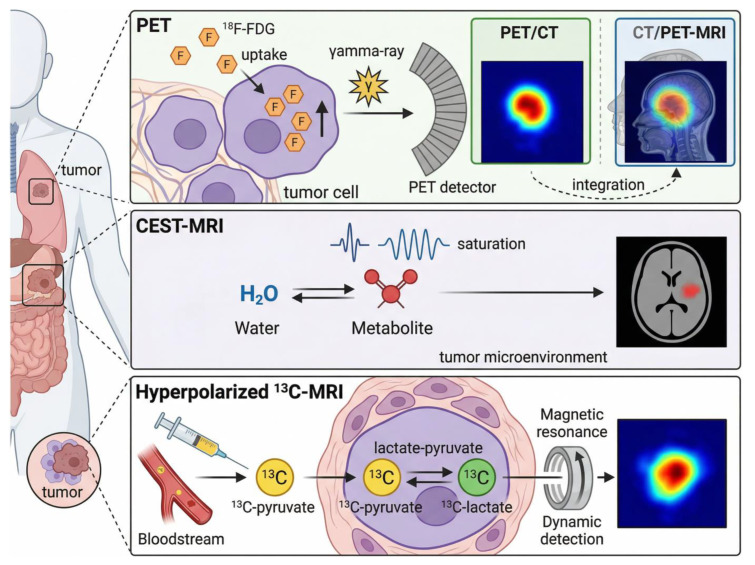
Schematic diagram of the principles of core functional and metabolic imaging technologies. Schematic diagram of the principles of core functional and metabolic imaging technologies. This figure illustrates the fundamental working mechanisms of key imaging modalities, including positron emission tomography (PET), PET/computed tomography (PET/CT), PET/magnetic resonance imaging (PET/MRI), chemical exchange saturation transfer MRI (CEST-MRI), and hyperpolarized 13C-MRI. Specifically, it depicts the molecular mechanism of radiotracer uptake (e.g., 18F-FDG) by tumor cells, the gamma-ray detection and image reconstruction process of PET, the complementary integration logic of metabolic information (from PET) and anatomical structure information (from CT/MRI), the proton chemical exchange saturation transfer principle of CEST-MRI, and the metabolic pathway visualization mechanism of hyperpolarized 13C-MRI (e.g., lactate-pyruvate conversion). This diagram synthesizes the core technical logic to facilitate the understanding of non-imaging professionals (e.g., clinical oncologists and basic researchers) on the functional characteristics of different imaging modalities.

In summary, PET technology’s strength lies in its ability to non-invasively capture functional tumor biology, but its clinical utility is contingent on tracer specificity, metric standardization, and appropriate integration with hybrid modalities. Ongoing research focuses on resolving these limitations through novel tracer development (e.g., dual-targeted agents), AI-driven image normalization, and multi-center validation of response thresholds—critical steps to solidify PET’s role in precision oncology workflows [8]. To intuitively illustrate the core technical logic and integration framework of functional/metabolic imaging, Figure 1 synthesizes the key principles of PET, PET/CT, PET/MRI, CEST-MRI, and hyperpolarized ^13^C-MRI, with emphasis on their clinical trade-offs and unresolved challenges [8]. To intuitively illustrate the working mechanisms of core functional/metabolic imaging techniques and their integration logic, Figure 1 summarizes the key principles of PET, PET/CT, PET/MRI, CEST-MRI, and hyperpolarized 13C-MRI.

### 2.2. Commonly Used Metabolic and Functional Imaging Agents

The landscape of cancer imaging has been significantly transformed by the advent of various metabolic and functional imaging agents, which provide crucial insights into tumor biology and treatment response (Table 1). Among these, 18F-fluorodeoxyglucose (18F-FDG) remains the most widely utilized radiotracer. It serves as a marker for glucose metabolism, reflecting the heightened metabolic activity characteristic of many tumors. This tracer is particularly valuable in the detection and monitoring of various malignancies, including lung, breast, and colorectal cancers. Its ability to visualize areas of increased glucose uptake allows clinicians to assess tumor viability and response to therapies effectively. However, the specificity of 18F-FDG can be compromised due to physiological uptake in normal tissues, leading to potential false positives. Notably, ^18^F-FDG is the only tracer in Table 1 with fully established clinical decision thresholds for treatment response (e.g., a ≥30% decrease in SUVmax for partial metabolic response in lymphoma), and it is the only agent approved for routine clinical use across all major oncology societies; all other tracers remain in varying stages of clinical validation or investigational use. Despite these limitations, its widespread application in clinical practice underscores its significance in oncological imaging [2].

Another promising agent is 18F-fluorothymidine (18F-FLT), which is specifically designed to reflect cellular proliferation by targeting thymidine metabolism. This tracer is particularly useful for early response monitoring in various cancers, as it allows for the assessment of changes in tumor growth rates in response to treatment. Unlike 18F-FDG, which primarily indicates metabolic activity, 18F-FLT provides direct insights into cellular proliferation, making it a valuable tool for evaluating the effectiveness of therapies aimed at inhibiting tumor growth. Research has shown that 18F-FLT PET imaging can detect early treatment responses before significant changes in tumor size become apparent on conventional imaging modalities, thereby facilitating timely therapeutic adjustments [11]. However, ^18^F-FLT suffers from inter-scanner variability in PI measurement of up to 25%, primarily due to differences in tracer injection dose and acquisition time windows, which has hindered the development of standardized clinical decision thresholds.

In addition to the established tracers, novel imaging agents are being developed to enhance our understanding of the tumor microenvironment and immune responses. One such agent is 18F-VC701, which targets immune-related molecules to reveal changes in the tumor immune microenvironment. A critical challenge in the interpretation of ^18^F-VC701 and other immune-targeted tracers is the metabolic overlap between tumor cells and immune infiltrates—both activated T cells and tumor cells exhibit increased glucose metabolism and thymidine uptake, leading to potential confounding of imaging signals and difficulty in distinguishing tumor-specific vs. immune cell-specific tracer accumulation. This tracer is particularly relevant in the context of immunotherapy, where understanding the immune landscape of tumors can guide treatment decisions and predict patient outcomes. By providing insights into the interactions between tumors and the immune system, 18F-VC701 represents a significant advancement in functional imaging, potentially leading to more personalized and effective treatment strategies for cancer patients [6].

The integration of these metabolic and functional imaging agents into clinical practice not only enhances our ability to diagnose and monitor cancer but also paves the way for the development of novel therapeutic approaches. As research continues to evolve, the exploration of new tracers and imaging modalities will likely lead to improved patient outcomes through more accurate assessments of tumor behavior and treatment efficacy. The ongoing advancements in imaging technology, coupled with the development of targeted tracers, hold great promise for the future of oncology, enabling clinicians to tailor treatment plans based on individual patient responses and tumor characteristics [12].

### 2.3. Multimodal Imaging Fusion Technology

The integration of various imaging modalities, particularly the fusion of Positron Emission Tomography (PET) with Magnetic Resonance Imaging (MRI) and Computed Tomography (CT), has significantly enhanced the accuracy of tumor localization and the completeness of functional information. This multimodal approach leverages the strengths of each imaging technique, allowing for a more comprehensive assessment of tumor characteristics (Table 2). PET provides metabolic information through the detection of radiotracers, which can indicate areas of increased cellular activity typically associated with malignancies. On the other hand, MRI offers superior soft tissue contrast and detailed anatomical visualization, while CT provides high-resolution images of the tumor’s structural integrity and surrounding anatomy. The combination of these modalities enables clinicians to obtain a more holistic view of the tumor, facilitating better diagnosis, staging, and treatment planning. For instance, in neuroendocrine tumors, a multimodal imaging approach can lead to improved detection rates and more precise localization of lesions, ultimately guiding more effective therapeutic interventions [13]. Furthermore, the integration of imaging data with clinical parameters has shown promise in improving the predictive accuracy of treatment responses, thereby aiding in personalized medicine strategies.

In addition to traditional imaging modalities, the incorporation of molecular techniques, such as single-cell RNA sequencing, allows for a deeper integration of imaging data with molecular characteristics of tumors. Imaging-omics integration is a conceptually attractive approach, but its current clinical translational maturity remains at a preclinical to early-phase clinical level (Level 1–2). To date, no imaging-omics signature has been validated for routine clinical decision-making, and the majority of studies are single-center, small-cohort analyses with limited external validation. This innovative approach facilitates the exploration of the tumor microenvironment and the identification of specific molecular signatures associated with treatment responses or resistance. A major risk in imaging-omics correlation is overfitting, driven by the high dimensionality of radiomic features (often thousands of features per image) relative to the small sample size of most multi-omics studies; this risk is compounded by false discovery due to multiple hypothesis testing without appropriate statistical correction (e.g., Bonferroni correction or false discovery rate (FDR) control). By correlating imaging findings with molecular data, researchers can uncover the underlying biological mechanisms driving tumor behavior and treatment efficacy. For example, studies have demonstrated that combining imaging with molecular profiling can enhance the understanding of tumor heterogeneity and inform more targeted therapeutic strategies, thus improving patient outcomes [17]. To mitigate overfitting and false discovery, the field has begun to adopt rigorous statistical approaches including feature selection (e.g., LASSO regression), cross-validation (e.g., k-fold cross-validation), and external validation in independent cohorts—though these practices are not yet standardized in imaging-omics research. The ability to visualize both anatomical and functional aspects of tumors alongside their molecular characteristics represents a significant advancement in the field of oncology, paving the way for more effective and individualized treatment regimens.

Moreover, the ongoing development of advanced imaging technologies, such as hybrid imaging systems that combine PET/MRI or PET/CT, continues to push the boundaries of multimodal imaging. These systems not only provide complementary information but also enhance the overall sensitivity and specificity of cancer detection. The integration of artificial intelligence (AI) and machine learning algorithms into these imaging modalities further enhances their capabilities, allowing for automated image analysis, improved diagnostic accuracy, and the potential for real-time monitoring of treatment responses [18]. A critical distinction in AI-driven radiomic analysis is between statistically significant radiomic features and clinically actionable ones: statistically significant features exhibit a robust correlation with a molecular or clinical endpoint in a study cohort, but clinically actionable features additionally have a clear, reproducible association with treatment decision-making or patient outcome, and are robust to inter-scanner/protocol variability. Most radiomic features identified to date are statistically significant but not clinically actionable, due to their lack of reproducibility and unclear biological relevance. As the field of multimodal imaging evolves, it is becoming increasingly evident that the fusion of imaging technologies with molecular insights will play a pivotal role in the future of cancer diagnosis and therapy, ultimately leading to improved patient care and outcomes.

## 3. Application of Functional and Metabolic Imaging in Early Response to Tumor Treatment

### 3.1. The Role of Metabolic Imaging in Predicting Response to Radiotherapy and Chemotherapy

Metabolic imaging, particularly through the use of positron emission tomography (PET) with fluorine-18-fluorothymidine (18F-FLT), has emerged as a pivotal tool in assessing the early responses of tumors to radiotherapy and chemotherapy. The ability of 18F-FLT PET to reflect tumor cell proliferation provides a significant advantage over traditional imaging modalities that primarily focus on anatomical changes. This is particularly relevant in the context of cancer treatment, where early detection of treatment efficacy can significantly influence clinical decision-making and patient outcomes. Studies have demonstrated that 18F-FLT PET can detect metabolic changes indicative of tumor response much earlier than conventional imaging techniques, which often rely on anatomical volume changes that may take longer to manifest. For instance, in patients undergoing treatment for myeloid sarcoma, significant correlations were found between the metabolic response measured by 18F-FLT PET and the clinical outcomes, suggesting that early metabolic responses could serve as predictive markers for treatment efficacy [19].

Moreover, the superiority of metabolic changes over anatomical volume changes is underscored by various studies that have shown how metabolic imaging can significantly shorten the response time window. In the context of non-Hodgkin lymphoma, interim PET scans performed after chemotherapy have been shown to provide critical insights into treatment effectiveness, with metabolic remission correlating with improved progression-free survival [20]. This highlights the potential for metabolic imaging to not only predict responses but also to guide subsequent treatment strategies, allowing for more personalized and adaptive therapeutic approaches. Notably, ^18^F-FDG PET is the only metabolic imaging modality with a clinically actionable decision threshold for lymphoma: a negative interim PET scan (Deauville score 1–3) is associated with a >80% progression-free survival rate and warrants continuation of standard therapy, while a positive scan (Deauville score 4–5) indicates the need for treatment escalation—this is the gold standard for imaging-guided decision-making in chemotherapy for lymphoma and the only such threshold with Level 3 clinical validation.

The predictive capabilities of metabolic imaging extend beyond mere response assessment; they also play a crucial role in identifying patients at risk for treatment resistance. For example, in locally advanced cervical cancer, metabolic tumor volume (MTV) and total lesion glycolysis (TLG) measured through PET imaging were found to correlate with treatment outcomes, enabling the identification of patients who may require more aggressive treatment strategies [21]. However, MTV and TLG exhibit greater inter-protocol variability than SUVmax (up to 40% across institutions), primarily due to differences in tumor segmentation algorithms, which has hindered the development of standardized clinical decision thresholds for these metrics in most tumor types. This ability to stratify patients based on metabolic imaging findings can lead to more tailored therapies, potentially improving overall survival rates.

In summary, metabolic imaging, particularly with 18F-FLT PET, represents a transformative advancement in the field of oncology, offering a dynamic and sensitive approach to monitoring treatment responses. Its ability to detect early metabolic changes not only enhances the predictive accuracy of treatment efficacy but also facilitates more personalized treatment regimens. As ongoing research continues to validate and refine the applications of metabolic imaging in various cancer types, it is poised to become an integral component of modern oncological practice, ultimately improving patient outcomes through more informed clinical decision-making.

### 3.2. Imaging Biomarkers and Treatment Sensitivity Associations

The heterogeneity of tumor cell populations presents a significant challenge in cancer treatment, particularly regarding the differential metabolic characteristics exhibited by these cells. Imaging biomarkers have emerged as crucial tools in distinguishing between sensitive and resistant cancer cell populations. For instance, various imaging modalities, including positron emission tomography (PET) and magnetic resonance imaging (MRI), can provide real-time insights into the metabolic activity of tumors. These imaging techniques can reveal distinct metabolic profiles that correlate with treatment sensitivity, allowing clinicians to tailor therapeutic strategies accordingly. In particular, studies have shown that changes in metabolic imaging parameters can predict treatment responses, as seen in breast cancer patients undergoing chemotherapy, where specific imaging biomarkers indicated varying degrees of sensitivity to treatment [22]. Furthermore, the ability to visualize metabolic alterations in real-time has allowed for the identification of resistant cell subpopulations that may not respond to standard therapies, emphasizing the importance of integrating imaging biomarkers into routine clinical practice for personalized cancer treatment [23]. Notably, imaging-derived heterogeneity outperforms biopsy-based molecular heterogeneity assessment in three key aspects: (1) spatial comprehensiveness—imaging captures the entire tumor mass, avoiding sampling bias from single-site biopsies; (2) temporal dynamics—imaging can be repeated serially to monitor changes in heterogeneity during treatment, while repeated biopsies are invasive and clinically impractical; (3) functional relevance—imaging measures functional heterogeneity (e.g., metabolic, proliferative), which is more directly linked to treatment response than the genetic heterogeneity measured by biopsy, which may include non-functional genetic alterations.

Moreover, the relationship between imaging parameters and molecular characteristics, such as gene expression profiles and immune cell infiltration, has been increasingly recognized. Imaging biomarkers can be correlated with specific genetic alterations or the presence of immune cells within the tumor microenvironment, thereby guiding precision medicine approaches. For example, in hepatocellular carcinoma (HCC), imaging biomarkers derived from computed tomography and MRI have been linked to the expression of various molecular markers, including alpha-fetoprotein (AFP) and programmed death-ligand 1 (PD-L1), which are indicative of tumor aggressiveness and immune evasion [23]. A key challenge in this correlation is accounting for metabolic overlap between tumor cells and immune infiltrates: PD-L1-positive immune cells exhibit increased glucose metabolism, leading to ^18^F-FDG uptake that can confound the assessment of tumor-specific metabolic activity and treatment response. This association underscores the potential of imaging biomarkers to provide comprehensive insights into the biological behavior of tumors, facilitating the identification of patients who may benefit from specific targeted therapies. Furthermore, the integration of imaging data with genomic and immunological profiles can enhance the predictive accuracy of treatment responses, thereby optimizing therapeutic decision-making [24].

In conclusion, the evolving landscape of imaging biomarkers in cancer treatment highlights their pivotal role in assessing treatment sensitivity and guiding personalized therapeutic strategies. By elucidating the intricate relationships between tumor metabolism, genetic alterations, and immune responses, imaging biomarkers can significantly improve patient stratification and treatment outcomes. As research continues to advance in this field, the incorporation of imaging biomarkers into clinical practice will likely become a standard approach for enhancing the efficacy of cancer therapies and overcoming treatment resistance.

### 3.3. Dynamic Imaging Monitoring and Treatment Adjustment

**Figure 2:**
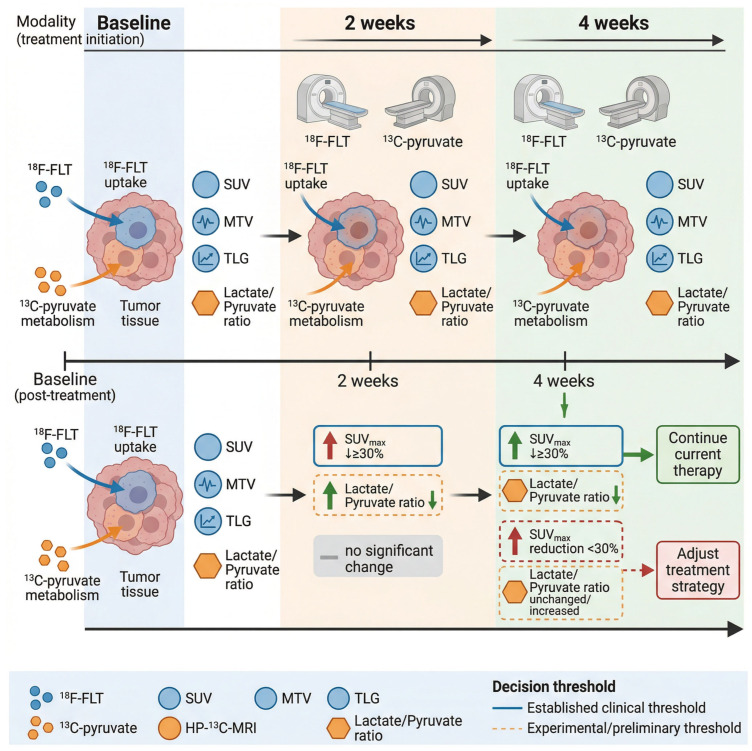
Flow diagram of imaging monitoring for early tumor treatment response. Flow diagram of imaging monitoring for early tumor treatment response. Guided by a time axis, this figure outlines the dynamic monitoring process from treatment initiation (baseline period) to early evaluation (2 weeks and 4 weeks post-treatment). It details the imaging techniques selected at different stages (e.g., 18F-FLT PET for assessing proliferation changes after radiotherapy/chemotherapy, hyperpolarized 13C-MRI for evaluating metabolic reprogramming after targeted therapy), core imaging metrics (standardized uptake value [SUV], metabolic tumor volume [MTV], total lesion glycolysis [TLG], and lactate/pyruvate ratio), the correlation between metric changes and treatment response (e.g., a ≥30% decrease in SUV indicates effective treatment), and the treatment adjustment pathway based on imaging results (maintaining the regimen for effective response and switching therapeutic strategies for ineffective response). This flow diagram provides an operable clinical reference for clinicians to implement personalized dynamic monitoring and treatment optimization.

Dynamic imaging monitoring has emerged as a pivotal strategy in the management of cancer treatment, allowing for timely detection of inadequate therapeutic responses and facilitating necessary adjustments to treatment regimens (Figure 2). Continuous imaging modalities, such as positron emission tomography (PET) and magnetic resonance imaging (MRI), enable clinicians to visualize metabolic changes and tumor responses in real-time, thus providing critical insights into the effectiveness of ongoing therapies. For instance, the use of hyperpolarized carbon-13 MRI has been shown to assess metabolic alterations in tumors, allowing for early detection of treatment responses that may not yet be evident through conventional imaging techniques that focus primarily on tumor size [14]. This capability is particularly crucial in cancers known for their rapid progression, such as pancreatic ductal adenocarcinoma, where early adjustments to treatment can significantly impact patient outcomes. Moreover, the integration of metabolic imaging biomarkers, such as lactate-to-pyruvate ratios, can provide a more nuanced understanding of tumor biology and treatment efficacy, enabling clinicians to tailor therapeutic approaches based on individual patient responses [25]. Notably, the lactate-to-pyruvate ratio measured by hyperpolarized ^13^C-MRI is a purely investigational biomarker with no established clinical decision thresholds, due to extreme inter-center variability driven by differences in hyperpolarizer equipment and tracer synthesis protocols.

In conjunction with imaging techniques, the incorporation of clinical indicators further enhances the personalization of treatment management. For example, the evaluation of circulating tumor DNA (ctDNA) has been demonstrated to offer insights into tumor burden and treatment response, allowing for the monitoring of disease progression and recurrence risk in real-time [26]. This approach not only aids in identifying patients who may benefit from intensified treatment strategies but also helps to avoid unnecessary exposure to ineffective therapies. The combination of imaging data with clinical parameters fosters a comprehensive understanding of each patient’s unique disease trajectory, thereby enhancing the precision of treatment adjustments.

Furthermore, the utilization of advanced imaging techniques in conjunction with biomarkers can significantly improve the stratification of patients for specific therapies, particularly in the context of targeted and immunotherapies. For instance, the dynamic assessment of immune cell activity through non-invasive imaging methods can provide critical information regarding patient responsiveness to immunotherapy, allowing for timely modifications to treatment plans based on real-time efficacy [27]. Again, metabolic overlap between tumor and immune cells remains a key confounder in this assessment: activated immune cells can lead to increased tracer uptake that may be misinterpreted as treatment resistance, while reduced immune cell uptake may reflect either successful immunotherapy or immune exhaustion. Such strategies not only optimize treatment outcomes but also minimize the risk of adverse effects associated with ineffective therapies, aligning with the overarching goals of precision oncology.

In conclusion, dynamic imaging monitoring combined with clinical indicators plays a crucial role in the early assessment of treatment responses and the adjustment of therapeutic strategies in oncology. By leveraging advanced imaging modalities and integrating them with clinical data, healthcare providers can enhance the personalization of cancer treatment, ultimately leading to improved patient outcomes and a more efficient allocation of healthcare resources. As the field of cancer treatment continues to evolve, the incorporation of these innovative approaches will be essential in addressing the complexities of tumor behavior and therapeutic resistance.

## 4. Research Progress on Functional and Metabolic Imaging in Tumor Drug Resistance Assessment

### 4.1. Imaging Manifestations of Tumor Heterogeneity and Resistance Mechanisms

The imaging of tumor heterogeneity has become a crucial aspect in understanding the mechanisms underlying treatment resistance in cancer therapy. Tumors are composed of diverse cellular populations, each exhibiting unique metabolic profiles and responses to therapeutic interventions. Recent studies utilizing advanced imaging techniques, such as multi-isotope imaging mass spectrometry (MIMS), have revealed significant metabolic heterogeneity within tumors. For instance, MIMS has demonstrated that subpopulations of tumor cells utilize different metabolic substrates, such as glucose and glutamine, which correlates with their proliferative capacity and therapeutic resistance [28]. This metabolic heterogeneity suggests the presence of drug-resistant cell populations that can survive treatment by adapting their metabolic pathways, thereby complicating therapeutic outcomes. Moreover, imaging technologies such as mass spectrometry imaging (MSI) have been instrumental in profiling the spatial distribution of metabolites and proteins within tumor microenvironments, highlighting areas of immune evasion and metabolic reprogramming that contribute to tumor progression and treatment failure [29]. A critical advantage of imaging-derived metabolic heterogeneity over biopsy-based molecular heterogeneity is its ability to link spatial metabolic patterns to treatment resistance in real-time; for example, imaging can identify hypoxic tumor regions (a key driver of resistance) that are not sampled by a standard biopsy, and monitor changes in these regions during hypoxia-sensitizing therapy—something that biopsy-based methods cannot achieve due to their static and localized nature.

The spatial analysis of tumor microenvironments using imaging techniques has also provided insights into the mechanisms of immune escape and metabolic alterations associated with resistance. For example, in the context of non-small cell lung cancer (NSCLC), MRI-based assessments have been developed to quantify tumor heterogeneity through the Tumor Heterogeneity Index (THI), which correlates significantly with tumor stages and treatment responses [15]. This index allows for a more nuanced understanding of how intratumoral heterogeneity can influence the efficacy of therapies, as heterogeneous tumors may harbor resistant cell populations that are less susceptible to conventional treatments. Furthermore, the integration of imaging modalities with molecular profiling has enhanced our understanding of the tumor microenvironment’s role in mediating resistance. For instance, the use of oxygen saturation imaging has elucidated the relationship between hypoxic tumor regions and the expression of resistance-related proteins, revealing how metabolic stress can drive tumor adaptation and survival [30]. THI is an investigational biomarker with Level 2 clinical validation in NSCLC; while it exhibits statistically significant correlation with treatment resistance, it is not yet clinically actionable due to inter-scanner variability in MRI sequence parameters (e.g., T2-weighted vs. contrast-enhanced T1) that affect index calculation.

Additionally, the application of advanced imaging techniques has enabled the visualization of cellular interactions within the tumor microenvironment, particularly between cancer cells and stromal components. Studies have shown that interactions between tumor-associated macrophages and cancer cells can significantly affect drug responses, as these immune cells can either promote or inhibit tumor growth depending on their metabolic states [31]. The ability to visualize these interactions in real-time using imaging technologies such as two-photon microscopy allows researchers to dissect the complex dynamics of tumor heterogeneity and its implications for therapy resistance. This level of detail is crucial for developing targeted therapies that can effectively address the diverse cellular populations within tumors.

In conclusion, the imaging manifestations of tumor heterogeneity provide valuable insights into the underlying mechanisms of treatment resistance. By employing advanced imaging techniques, researchers can elucidate the metabolic diversity of tumor cells, the spatial dynamics of the tumor microenvironment, and the interactions between cancer cells and stromal components. These insights are essential for the development of personalized treatment strategies that can effectively target the heterogeneous nature of tumors and improve therapeutic outcomes for patients. As imaging technologies continue to evolve, their integration with molecular profiling will undoubtedly enhance our understanding of cancer biology and pave the way for more effective interventions against drug-resistant tumors.

### 4.2. The Association Between the Immune Microenvironment and Metabolic Imaging

The interplay between the immune microenvironment and metabolic imaging has emerged as a key area of interest in cancer research, particularly in understanding tumor progression and therapeutic responses. Metabolic imaging, particularly with tracers like 18F-VC701, provides insights into the metabolic activity of immune cells within the tumor microenvironment. This imaging modality can reflect the activation states of various immune cell populations, revealing critical mechanisms of immune evasion that tumors exploit. The most significant challenge in this association is the metabolic overlap between tumor cells and immune infiltrates: both tumor cells and activated immune cells (e.g., effector T cells, macrophages) exhibit increased glycolysis, glutaminolysis, and nucleotide synthesis, leading to overlapping tracer uptake for metabolic and proliferation-based imaging agents (^18^F-FDG, ^18^F-FLT). This overlap confounds the interpretation of imaging signals, as increased tracer uptake may indicate either tumor progression or an effective anti-tumor immune response, and decreased uptake may reflect either treatment efficacy or immune suppression. For instance, studies have shown that metabolic reprogramming in tumors can lead to altered immune cell infiltration and activation, thus impacting the efficacy of immunotherapies [32]. To address this confounder, researchers have developed two complementary strategies: (1) dual-tracer imaging with agents targeting tumor-specific markers (e.g., ^68^Ga-FAPI) and immune-specific markers (e.g., ^18^F-VC701) to spatially resolve the two cell populations; and (2) radiomic feature analysis to distinguish the distinct spatial patterns of tracer accumulation (e.g., focal uptake in tumor cells vs. diffuse uptake in immune infiltrates). Both strategies remain investigational and add significant cost and complexity to clinical imaging workflows.

Moreover, the findings from metabolic imaging studies support the design of combination therapies that integrate immune modulation with metabolic intervention. For example, the use of 18F-FDG PET imaging has been instrumental in assessing glucose metabolism not only in tumor cells but also in immune cells, providing a comprehensive view of the metabolic dynamics within the tumor microenvironment [33]. This dual perspective is crucial, as it highlights how metabolic alterations in cancer cells can suppress immune responses, thereby fostering an immunosuppressive microenvironment conducive to tumor growth and metastasis. Consequently, leveraging metabolic imaging can guide the development of therapeutic strategies that aim to enhance immune cell activation while simultaneously targeting the metabolic vulnerabilities of cancer cells.

The implications of these associations extend to clinical practice, where metabolic imaging can serve as a predictive biomarker for treatment responses to immunotherapy. For instance, increased metabolic activity observed in immune cells via PET imaging has been correlated with better responses to immune checkpoint inhibitors, suggesting that metabolic imaging could be utilized to stratify patients based on their likelihood of benefiting from such therapies [34]. Notably, this correlation is based on Level 2 clinical validation data; no immune-targeted imaging tracer has yet been validated for routine clinical stratification of immunotherapy patients, and all current approaches remain investigational. This predictive capability underscores the potential of metabolic imaging to inform personalized treatment approaches, allowing for timely adjustments in therapeutic strategies based on real-time assessments of the immune microenvironment.

In summary, the relationship between the immune microenvironment and metabolic imaging is multifaceted and holds significant promise for advancing cancer treatment (Table 3). By elucidating the metabolic profiles of immune cells and their interactions with tumor cells, metabolic imaging not only enhances our understanding of tumor biology but also paves the way for innovative therapeutic strategies that combine metabolic and immunological interventions. As research continues to evolve in this domain, the integration of metabolic imaging into clinical practice could revolutionize how we approach cancer treatment, making it more personalized and effective [35].

### 4.3. Long Non-Coding RNAs and Imaging Studies on Drug Resistance

Long non-coding RNAs (lncRNAs) have emerged as crucial regulators in various biological processes, including tumorigenesis and drug resistance in cancer therapy. Recent advancements in single-cell RNA sequencing have enabled researchers to correlate imaging features with lncRNA expression profiles, thereby elucidating the molecular mechanisms underlying drug resistance. For instance, studies have demonstrated that specific lncRNAs, such as LINC01123, play a significant role in the chemoresistance of non-small cell lung cancer (NSCLC) by interacting with key signaling pathways. The use of imaging techniques like 68Ga-FAPI PET/CT has allowed for the visualization of cancer-associated fibroblasts (CAFs) and their influence on lncRNA expression, providing insights into the tumor microenvironment’s role in therapeutic resistance [9]. By integrating imaging data with lncRNA expression profiles, researchers can identify potential biomarkers for drug resistance, facilitating the development of targeted therapies. For example, the identification of lncRNAs that are upregulated in resistant cancer cells can aid in the selection of patients who are likely to benefit from specific treatment regimens, thus personalizing cancer therapy [10]. All imaging-lncRNA correlation studies to date are Level 1 (preclinical/early-phase) with no clinical actionable biomarkers; the primary challenge is the high risk of overfitting due to the small sample size of lncRNA studies and high dimensionality of imaging features.

Furthermore, the application of imaging modalities in conjunction with lncRNA studies can enhance the understanding of how these non-coding RNAs modulate the tumor microenvironment and contribute to therapeutic resistance. For instance, glioblastoma multiforme (GBM) research has highlighted the role of tumor-derived exosomes containing lncRNAs, which can influence immunomodulation and drug resistance [37]. Imaging techniques can track these exosomes, providing a non-invasive means to monitor changes in lncRNA expression associated with treatment response. This approach not only aids in the early diagnosis of GBM but also in the assessment of therapeutic efficacy, as changes in lncRNA levels can serve as biomarkers for monitoring patient responses to chemotherapy [36]. Imaging tracking of exosomes remains a purely investigational technique with no clinical validation, due to the lack of specific, high-sensitivity tracers for exosome-associated lncRNAs.

Moreover, the integration of imaging modalities with lncRNA profiling can significantly advance the field of precision medicine. By identifying lncRNAs that correlate with specific imaging characteristics, clinicians can develop more effective treatment strategies tailored to individual patients. For example, the use of magnetic resonance molecular imaging (MRMI) has been explored to assess the efficacy of combining lncRNA-targeted therapies with conventional chemotherapy in triple-negative breast cancer (TNBC) [36]. MRMI for lncRNA-targeted therapy assessment is a Level 1 investigational technique; while it exhibits statistically significant correlations with treatment efficacy in preclinical studies, it has not been validated in clinical cohorts and has no established decision thresholds. This combination approach not only enhances treatment outcomes but also provides a framework for understanding the complex interactions between lncRNAs and the tumor microenvironment, ultimately leading to improved patient management.

In conclusion, the intersection of lncRNA research and imaging studies represents a promising frontier in cancer therapy, particularly in understanding and overcoming drug resistance. By leveraging advanced imaging techniques alongside single-cell RNA sequencing, researchers can uncover the intricate relationships between lncRNA expression and tumor characteristics, paving the way for the identification of novel biomarkers and therapeutic targets. To advance clinical translation, future studies must address the risk of overfitting through large, multi-center cohorts, standardized imaging protocols, and rigorous statistical correction for multiple hypothesis testing. This integrative approach holds the potential to revolutionize cancer treatment paradigms, enabling more personalized and effective strategies for managing resistant tumors [38].

## 5. Clinical Application Cases and Multidisciplinary Integration Analysis

### 5.1. Imaging Applications in Glioblastoma Treatment

The management of glioblastoma (GBM), one of the most aggressive forms of brain cancer, has seen significant advancements through the integration of imaging techniques that enhance treatment personalization and efficacy. Recent studies have highlighted the role of imaging modalities in evaluating the effectiveness of combined therapies, such as radiotherapy and chemotherapy, alongside metabolic modulation agents like metformin. For instance, positron emission tomography (PET) imaging using amino acid tracers, such as O-[2-(18F)fluoroethyl]-L-tyrosine (FET), has emerged as a valuable tool for assessing tumor metabolism and response to treatment. This technique allows for the early detection of treatment effects, which is crucial for extending patient survival. A study demonstrated that radiomic features extracted from FET PET images could effectively predict treatment response by capturing metabolic changes in the tumor over time, thus guiding personalized therapeutic strategies [39]. Notably, FET PET radiomic features are statistically significant predictors of GBM treatment response but not yet clinically actionable, due to inter-scanner variability in PET acquisition parameters and lack of standardized feature selection algorithms.

Furthermore, the application of advanced imaging techniques has facilitated the identification of tumor heterogeneity, which is pivotal in understanding treatment resistance in GBM. The use of magnetic resonance imaging (MRI) combined with spectroscopy has provided insights into the biochemical landscape of tumors, allowing for the assessment of biomarkers such as MGMT promoter methylation status, which correlates with treatment outcomes. This integration of imaging and biochemical markers can enhance the accuracy of prognostic evaluations and therapeutic decisions [40]. MRI-based assessment of MGMT methylation is a Level 2 investigational biomarker; while it exhibits moderate correlation with MGMT status in clinical studies, it is not yet used for routine clinical decision-making due to lower accuracy compared to biopsy-based molecular testing. Moreover, the implementation of artificial intelligence (AI) and machine learning algorithms in imaging analysis has shown promise in predicting patient responses to therapies, thereby optimizing treatment plans and improving overall survival rates [41]. AI-driven MRI analysis for GBM prognosis is a Level 1 investigational technique with high overfitting risk in single-center studies; external validation in multi-center cohorts is ongoing.

In the context of personalized medicine, the early prediction of treatment efficacy through imaging can significantly influence clinical outcomes. For example, the use of radiomics to analyze preoperative MRI scans has been shown to correlate with early recurrence of glioblastoma, providing a non-invasive method for stratifying patients based on their likelihood of response to therapy [42]. These radiomic signatures for GBM recurrence prediction are statistically significant in single-center cohorts but lack clinical actionability, primarily due to poor reproducibility across different MRI scanners and sequence protocols, as well as the absence of standardized cut-off values for risk stratification. The ability to visualize tumor response dynamically allows clinicians to adapt treatment regimens promptly, thereby enhancing the therapeutic impact and minimizing unnecessary side effects.

Additionally, the exploration of novel imaging modalities, such as hyperspectral imaging and fluorescence-guided surgery, has opened new avenues for intraoperative tumor detection and delineation. These techniques can improve surgical precision by allowing real-time visualization of tumor margins, thus facilitating more complete resections while preserving critical brain functions [43]. Notably, fluorescence-guided surgery for GBM has achieved Level 2 clinical validation, with utility in specialized neuro-oncology centers for improving gross total resection rates; however, it remains investigational for routine use due to high costs of fluorescent tracers and the need for dedicated intraoperative imaging equipment, which limits accessibility in community hospitals. The combination of these advanced imaging techniques with emerging therapeutic strategies, such as immunotherapy and targeted drug delivery systems, holds the potential to revolutionize the treatment landscape for glioblastoma, ultimately leading to better patient outcomes and survival rates [44]. Despite this potential, the integration of novel imaging with emerging GBM therapies is currently limited to early-phase clinical trials, with no established clinical decision frameworks for imaging-guided selection of immunotherapy or targeted therapy regimens.

In conclusion, the integration of imaging applications in glioblastoma treatment represents a significant advancement in the field of neuro-oncology. By enabling early prediction of treatment responses, guiding personalized therapies, and improving surgical outcomes, these imaging modalities are essential tools in the ongoing battle against this formidable disease. To translate investigational imaging modalities into routine clinical practice for GBM, key steps include harmonizing imaging protocols across centers, establishing validated decision thresholds for imaging biomarkers, and conducting cost-effectiveness analyses to justify the adoption of high-cost technologies in resource-constrained settings. Continued research and innovation in imaging technologies will be crucial in enhancing the efficacy of glioblastoma treatments and improving the quality of life for affected patients.

### 5.2. Multimodal Imaging and Single-Cell Omics Joint Analysis

The integration of multimodal imaging and single-cell RNA sequencing (scRNA-seq) has emerged as a powerful approach to unravel the complexities of the tumor microenvironment and assess therapeutic resistance. While conceptually transformative, imaging-omics integration currently has a low level of clinical translational maturity—nearly all published studies are exploratory, single-center analyses with small sample sizes (*n* < 50 in most cases), and no imaging-omics signature has been validated for routine clinical decision-making or received regulatory approval. Traditional imaging techniques, while valuable, often provide limited insights into the heterogeneous cellular composition and functional states within tumors. In contrast, scRNA-seq allows for the detailed characterization of individual cell types, revealing their unique gene expression profiles and biological functions. By combining these modalities, researchers can achieve a more comprehensive understanding of how different cell populations interact within the tumor microenvironment and contribute to treatment responses. For instance, recent studies have demonstrated that spatial transcriptomics, when coupled with multiplexed imaging techniques, can map the distribution of various cell types and their corresponding transcriptomic profiles within the same tissue section. This integrative approach has proven crucial in identifying specific cellular interactions and signaling pathways that drive tumor progression and therapeutic resistance [45]. However, these spatial multi-omics imaging approaches are purely investigational (Level 1 validation), limited to preclinical and early-phase clinical research by the high cost of spatial transcriptomics assays, the need for specialized computational expertise for data integration, and the lack of standardized workflows for correlating imaging features with single-cell data. Moreover, the use of advanced imaging technologies such as mass spectrometry imaging (MSI) and fluorescence microscopy enables the visualization of spatially resolved metabolic and proteomic landscapes, further enriching the data obtained from scRNA-seq analyses [46].

A central challenge in imaging-omics joint analysis is mitigating the risks of overfitting and false discovery, which are amplified by the high dimensionality of both datasets: radiomic analysis typically generates thousands of quantitative features per image, while scRNA-seq profiles tens of thousands of genes per cell. These high-dimensional datasets are often paired with small patient cohorts, leading to model overfitting—where the integrated model performs well on the training data but fails to generalize to independent validation cohorts. To address this, the field has begun to adopt rigorous statistical and computational safeguards, including: (1) stringent feature selection (e.g., LASSO regression, mutual information filtering) to reduce radiomic and omics feature dimensionality to a biologically relevant subset; (2) nested k-fold cross-validation to avoid data leakage during model training; (3) correction for multiple hypothesis testing (e.g., FDR control, Bonferroni correction) to minimize false discovery of correlated imaging-omics features; and (4) external validation in geographically distinct, multi-center cohorts to confirm model generalizability. Despite these advances, such rigorous practices are not yet standardized in imaging-omics research, with many studies still relying on simple univariate correlation and single-fold cross-validation, which overestimates model performance and limits clinical translatability.

The insights gained from multimodal imaging and single-cell analyses support the development of multi-target combination therapy strategies. By elucidating the intricate cellular networks and pathways involved in tumorigenesis and therapy resistance, researchers can identify potential therapeutic targets that may not be apparent when using single modalities. For example, the identification of specific immune cell subsets that exhibit distinct transcriptional profiles in response to treatment can inform the design of immunotherapies tailored to individual patients [47]. However, the translation of these imaging-omics derived therapeutic targets to clinical trials remains in its infancy, as most identified targets are based on correlative rather than causal imaging-omics associations, and require further functional validation in preclinical models. Furthermore, integrating these modalities can reveal the dynamic changes in the tumor microenvironment over time, allowing for the identification of early biomarkers of treatment response or resistance. This is particularly relevant in the context of neoadjuvant therapies, where understanding the early cellular responses to treatment can guide subsequent therapeutic decisions [48].

**Figure 3:**
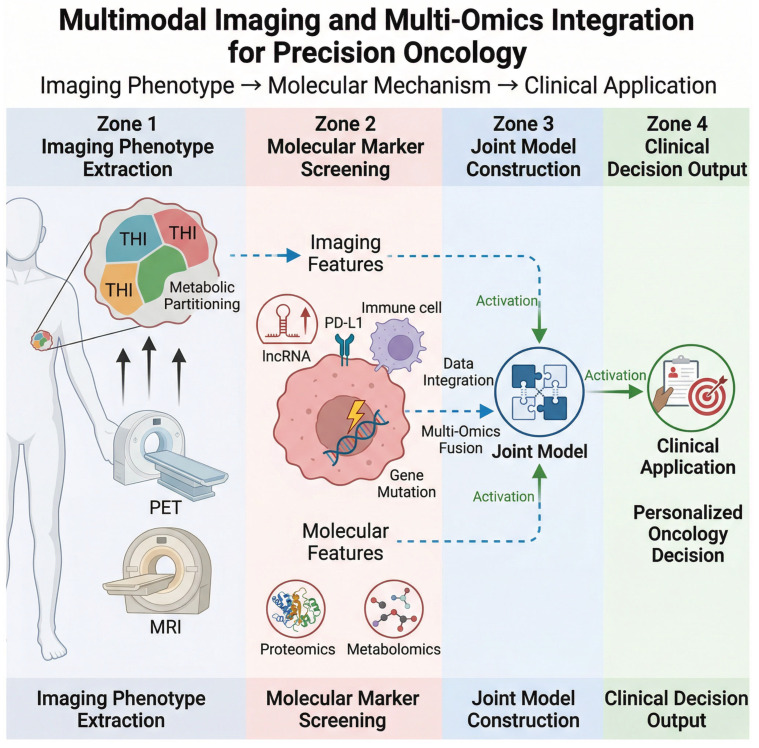
Schematic diagram of multimodal imaging and multi-omics integration analysis. Schematic diagram of multimodal imaging and multi-omics integration analysis. This figure illustrates the integration logic between multimodal imaging data (e.g., PET/MRI) and multi-omics data, including single-cell RNA sequencing, genomics, proteomics, and metabolomics. It explicitly presents the corresponding relationships between imaging phenotypes (e.g., Tumor Heterogeneity Index [THI], metabolic partitioning) and molecular characteristics (e.g., long non-coding RNA [lncRNA] expression, programmed death-ligand 1 [PD-L1] level, gene mutation), as well as the key steps of data integration: imaging feature extraction → molecular marker screening → joint model construction → clinical decision output. This diagram highlights the core argument of “multidisciplinary integration” in the review, clarifying the transmission path of “imaging phenotype → molecular mechanism → clinical application” and enhancing the systematic understanding of precision oncology driven by imaging-omics integration.

In summary, the joint analysis of multimodal imaging and single-cell omics represents a significant advancement in our ability to dissect the complexities of tumor biology and therapeutic responses. This integrative approach not only enhances our understanding of the tumor microenvironment but also paves the way for the development of more effective, personalized treatment strategies. As technology continues to evolve, the potential for these methodologies to inform clinical practice and improve patient outcomes will likely expand, underscoring the importance of continued research in this area [49]. To clarify the integration logic between multimodal imaging data and multi-omics (single-cell RNA sequencing, genomics, proteomics, metabolomics) and its translation to clinical practice, Figure 3 illustrates the key links and workflow.

### 5.3. Imaging-Driven Precision Treatment Decisions

The role of imaging biomarkers as clinical decision support tools is becoming increasingly vital in optimizing treatment selection and monitoring therapeutic efficacy in oncology. Imaging modalities, particularly molecular imaging techniques, provide critical insights into tumor biology, enabling the customization of treatment strategies tailored to individual patient profiles. For instance, positron emission tomography (PET) and magnetic resonance imaging (MRI) have demonstrated their utility in assessing metabolic activity and anatomical details of tumors, respectively. These imaging techniques facilitate the identification of specific therapeutic targets, allowing clinicians to select the most appropriate targeted therapies for patients based on the unique characteristics of their tumors. Moreover, imaging biomarkers can assist in monitoring treatment responses in real-time, providing valuable feedback that can guide adjustments in therapy. This dynamic approach not only enhances the precision of cancer treatment but also minimizes the risk of overtreatment and associated toxicities, ultimately leading to improved patient outcomes and quality of life [50].

In the context of clinical trials, imaging plays a pivotal role in evaluating therapeutic efficacy and identifying potential biomarkers for patient stratification. The integration of imaging data with clinical and genomic information can enhance the predictive power of clinical trials, allowing for more informed decisions regarding patient eligibility and treatment regimens. For example, the use of imaging biomarkers in early-phase clinical trials can help determine which patients are more likely to benefit from specific therapies, thereby streamlining the drug development process and reducing the time and resources required to bring new treatments to market. Additionally, the incorporation of advanced imaging techniques, such as radiomics and machine learning algorithms, is revolutionizing the landscape of precision medicine by enabling the extraction of complex data patterns from imaging studies. These patterns can provide insights into tumor heterogeneity and treatment resistance, further refining patient selection and therapeutic strategies [51].

Furthermore, the concept of theranostics, which combines diagnostic imaging with targeted therapy, exemplifies the potential of imaging-driven precision treatment decisions. By utilizing radiolabeled ligands that can both visualize tumors and deliver therapeutic agents, clinicians can achieve a more personalized approach to cancer treatment. This dual capability not only enhances the specificity of therapy but also allows for real-time monitoring of treatment responses, thereby facilitating timely adjustments to therapeutic plans based on individual patient needs. The successful application of theranostic agents, particularly in cancers such as prostate cancer, underscores the importance of integrating imaging into clinical decision-making processes [52].

In conclusion, the evolving landscape of imaging technologies and their integration into clinical practice are transforming the approach to cancer treatment. By leveraging imaging biomarkers as decision support tools, clinicians can optimize treatment selection, monitor therapeutic efficacy, and ultimately improve patient outcomes. The current clinical utility of imaging-driven precision oncology is centered on a small set of Level 3 validated biomarkers, with the vast majority of innovative imaging approaches remaining investigational. To expand this utility, future research must prioritize multi-center RCTs to demonstrate clinical utility, harmonization of imaging protocols to improve reproducibility, and the development of cost-effective workflows for integrating advanced imaging into routine oncology care. The continued advancement of imaging modalities, coupled with the development of novel therapeutic agents, holds great promise for enhancing the precision of cancer care and addressing the challenges associated with tumor heterogeneity and treatment resistance [53].

## 6. Challenges and Future Directions of Functional and Metabolic Imaging Technologies

### 6.1. Technical Limitations and Standardization Issues

The application of functional and metabolic imaging in tumor treatment response assessment and drug resistance evaluation has shown great promise; however, several technical limitations and standardization issues remain prevalent. One significant challenge is the specificity and sensitivity of tracers used in imaging modalities. Current tracers often lack the necessary specificity to differentiate between tumor and normal tissues effectively, leading to a high incidence of false positives and negatives. For instance, the metabolic activity of tumor cells can closely resemble that of normal cells, particularly in the context of increased background signals during imaging, which complicates the interpretation of results [54]. Enhancing the specificity of tracers is crucial to improve the accuracy of tumor detection and to minimize the risk of misdiagnosis, which could lead to inappropriate treatment decisions. Furthermore, advancements in imaging technologies, such as the development of proximity-enhanced functional imaging (PEFI), aim to address these limitations by improving the detection of metabolic differences between tumor and normal cells [55]. Despite these advancements, the need for ongoing research to refine tracer development and enhance their specificity remains critical.

Additionally, the standardization of imaging data acquisition and analysis poses a significant hurdle in achieving reliable results across different centers. This lack of standardization is the primary driver of inter-scanner and inter-protocol variability for core imaging biomarkers (SUV, MTV, TLG, THI), with variability rates ranging from 20 to 40% across institutions for most metrics—well above the 10–15% variability threshold required for clinical actionability. Variability in imaging protocols, including differences in equipment, settings, and analysis techniques, can lead to discrepancies in the interpretation of imaging data, hindering the ability to compare results across studies or institutions [7]. For example, the lack of standardized protocols for functional imaging techniques can result in variations in how parameters such as metabolic tumor volume and standardized uptake values are measured and reported. This inconsistency can complicate the assessment of treatment responses and the evaluation of drug resistance mechanisms, as observed in neuroendocrine tumors where molecular imaging has not yet established standardized response criteria [56]. To address these challenges, it is essential to develop comprehensive guidelines and best practices for imaging protocols that can be adopted universally. Collaborative efforts among researchers and institutions to establish consensus on imaging methodologies and data reporting standards will enhance the reliability and comparability of imaging studies, ultimately benefiting patient care and outcomes.

In summary, while functional and metabolic imaging holds significant potential for evaluating tumor treatment responses and drug resistance, addressing the technical limitations related to tracer specificity and the standardization of imaging practices is imperative. Key steps to overcome these challenges include: (1) the development of tumor-specific tracers with minimal off-target uptake to reduce false positives/negatives; (2) mandatory implementation of harmonized imaging protocols with regular quality control in all oncology centers; (3) the adoption of automated, AI-driven image analysis tools to eliminate human variability in segmentation and biomarker calculation; and (4) the establishment of central imaging core labs for multi-center studies to ensure consistent data analysis. Continued research and collaboration in these areas will pave the way for improved imaging techniques that can provide more accurate assessments of tumor biology, thereby guiding more effective treatment strategies for cancer patients.

### 6.2. Novel Tracers and Innovations in Imaging Technology

The development of novel tracers targeting new metabolic pathways and immune markers in tumors has significantly advanced the field of cancer imaging. These innovative tracers are designed to enhance the specificity and sensitivity of imaging modalities, enabling better detection and characterization of tumors. Despite these advances, the widespread adoption of novel tracers and advanced imaging technologies is hindered by a series of key technical bottlenecks, which are the primary barriers preventing translation from research to routine clinical use. For instance, recent research has focused on combining photodynamic therapy (PDT) with imaging technologies to create theranostic agents that can both diagnose and treat cancer simultaneously. Such agents utilize photosensitizers that are activated by specific wavelengths of light, allowing for targeted therapy while providing real-time imaging feedback on treatment effectiveness [57]. Additionally, the introduction of advanced molecular imaging techniques, such as fluorescence imaging, has improved the visualization of tumor metabolism and microenvironmental changes. These techniques utilize novel fluorescent probes that can specifically bind to tumor-associated antigens or metabolic markers, providing insights into tumor biology and treatment response [58]. Furthermore, the integration of artificial intelligence (AI) in imaging analysis has enhanced the ability to interpret complex imaging data, leading to improved diagnostic accuracy and predictive capabilities in assessing treatment outcomes [59].

Moreover, the exploration of novel imaging modalities, such as terahertz (THz) imaging and contrast-enhanced ultrasound, has opened new avenues for early cancer detection and monitoring. THz imaging, for example, leverages the unique optical properties of cancerous tissues to provide label-free, non-invasive imaging, which can be particularly useful in detecting early-stage tumors [60]. On the other hand, advancements in ultrasound technology, including the development of nanobubble contrast agents, have shown promise in enhancing the imaging of tumors and improving the delivery of therapeutics [61]. These nanobubbles can provide better contrast and resolution in ultrasound imaging, allowing for more accurate assessments of tumor characteristics and treatment responses. While these modalities offer unique advantages (e.g., low cost, no ionizing radiation), their adoption is limited by technical bottlenecks such as low spatial resolution (THz imaging) and poor deep tissue penetration (nanobubble ultrasound), which restrict their use to superficial tumors only.

Furthermore, the combination of molecular imaging with other diagnostic modalities, such as positron emission tomography (PET) and magnetic resonance imaging (MRI), has resulted in the development of multimodal imaging systems that can provide comprehensive insights into tumor biology. For instance, integrating PET with MRI allows for the simultaneous assessment of metabolic activity and anatomical structure, enhancing the overall diagnostic capability [62]. This multimodal approach is particularly beneficial in evaluating the tumor immune microenvironment, which is crucial for understanding treatment responses and resistance mechanisms [59].

In summary, the ongoing innovations in tracer development and imaging technologies are transforming cancer diagnostics and treatment monitoring. These advancements not only improve the precision of tumor characterization but also facilitate personalized treatment strategies by providing real-time insights into tumor behavior and treatment efficacy. To overcome the technical bottlenecks limiting widespread adoption, future research must prioritize: (1) the development of scalable, off-the-shelf novel tracers that can be distributed to community hospitals; (2) the miniaturization and cost-reduction in specialized equipment (e.g., portable hyperpolarizers, low-cost PET/MRI scanners); (3) the engineering of high-affinity, stable tracers with improved tumor-to-background SNR; and (4) the development of user-friendly, automated image analysis tools for advanced imaging modalities that require no specialized computational expertise. The future of cancer imaging will likely continue to evolve with the integration of novel tracers, advanced imaging techniques, and AI-driven analysis, ultimately enhancing patient outcomes through more accurate and timely interventions.

### 6.3. Multi-Omics Integration and Personalized Precision Medicine

A critical advancement in addressing tumor heterogeneity—the primary barrier to effective cancer therapy—lies in the complementary synergy between functional and metabolic imaging and state-of-the-art single-cell technologies, where each modality addresses inherent limitations of the other to provide a holistic characterization of the tumor microenvironment (TME). Functional imaging modalities including PET/MRI, CEST-MRI, and hyperpolarized ^13^C-MRI deliver macroscopic, spatially resolved insights into tumor metabolic activity, proliferative status, and microenvironmental features (e.g., hypoxia, stromal remodeling) at the tissue level, enabling non-invasive, real-time monitoring of TME dynamics in vivo. In contrast, single-cell omics technologies offer unparalleled high-resolution profiling of cellular heterogeneity, unraveling the molecular and functional diversity of cancer cells, immune cells, and stromal components within the TME at the single-cell level. Recent landmark single-cell studies have exemplified this power: a single-cell RNA sequencing utilized single-cell RNA sequencing to characterize melanoma tumor microenvironment heterogeneity and predict immunotherapy response [63]; another study has revealed the cell types that potentially affect ICIs and identified potential drugs by combining bulk sequencing and single-cell sequencing [64]. Integrating these macroscopic functional imaging phenotypes with cellular-level single-cell mechanistic insights creates a critical link between in vivo, clinically translatable imaging biomarkers and the underlying cellular and molecular events that govern tumor behavior and treatment response. This integrative approach transcends the limitations of standalone modalities: functional imaging can guide the spatial sampling of tumor regions for single-cell analysis, ensuring that high-resolution molecular profiling is anchored to clinically relevant, in vivo TME features, while single-cell technologies elucidate the cellular mechanisms that underpin imaging phenotypes (e.g., elevated ^68^Ga-FAPI uptake or increased Tumor Heterogeneity Index). For precision oncology, this synergy enables the translation of cellular-level mechanistic discoveries into clinically actionable imaging biomarkers: for example, single-cell identification of a subpopulation that drives chemoresistance (PMID: 38896289) can be correlated with ^68^Ga-FAPI PET imaging signals to develop non-invasive biomarkers for stratifying patients who may benefit from CAF-targeted therapies. Conversely, functional imaging detection of heterogeneous metabolic activity within a tumor can be interrogated via single-cell sequencing (PMID: 40092490) to identify the specific cellular subpopulations and metabolic pathways responsible for this heterogeneity, informing the design of personalized combination therapies that target these pathways. Ultimately, the integration of functional and metabolic imaging with single-cell technologies represents a paradigm shift in tumor heterogeneity research, bridging the macroscopic and cellular scales to advance the development of mechanism-driven, imaging-guided precision oncology strategies that directly address the root cause of treatment resistance in cancer.

The integration of multi-omics approaches, including imaging genomics, transcriptomics, proteomics, and metabolomics, presents a transformative opportunity to enhance the prediction of tumor treatment responses and the evaluation of therapeutic resistance. By constructing comprehensive tumor treatment response prediction models that incorporate imaging data alongside genomic and immunological profiles, researchers can better understand the complex interplay between tumor biology and therapeutic efficacy. Recent studies have demonstrated that multi-omics integration can reveal distinct molecular signatures associated with treatment outcomes, such as pathologic complete response (pCR) in breast cancer patients receiving immunotherapy combined with targeted therapies. For example, a multiomic factor analysis identified key biomarkers related to immune response and tumorigenesis, which were significantly associated with pCR rates, indicating that patients with higher multiomic factor scores had substantially improved treatment outcomes [65]. This underscores the importance of leveraging diverse biological data to develop predictive models that can guide personalized treatment strategies, ultimately leading to enhanced patient outcomes. However, these multi-omics prediction models often suffer from overfitting due to the high dimensionality of the combined imaging and omics data, and most have not been validated in independent, multi-center cohorts—limiting their clinical translatability.

Furthermore, the dynamic management of cancer patients can be significantly improved through the widespread application of advanced imaging technologies in clinical pathways. By employing liquid biopsy techniques to capture circulating tumor DNA (ctDNA) and circulating tumor cells (CTCs), clinicians can monitor real-time changes in tumor dynamics and treatment responses. This non-invasive approach allows for the continuous assessment of tumor evolution and therapeutic efficacy, facilitating timely adjustments in treatment plans according to individual patient needs. For instance, liquid biopsy analyses have shown potential in identifying minimal residual disease and predicting relapse, making it a valuable tool for personalized medicine [66]. The integration of imaging technology with liquid biopsy multi-omics data is a promising emerging approach, as it combines the spatial, functional insights of imaging with the molecular, real-time insights of liquid biopsies. This integration is currently in Level 2 clinical validation, with utility in specialized centers for monitoring minimal residual disease in breast and prostate cancer, but remains investigational for routine use due to the high cost of combined imaging and liquid biopsy testing. The integration of imaging technology with multi-omics data not only enhances the precision of cancer diagnostics but also empowers clinicians to implement tailored treatment regimens that align with the unique molecular characteristics of each patient’s tumor.

Moreover, the advancement of artificial intelligence (AI) and machine learning in processing and analyzing multi-omics data is paving the way for more sophisticated predictive models that can inform clinical decision-making. AI-driven algorithms can synthesize vast amounts of multi-omics data to identify biomarkers associated with treatment responses and resistance mechanisms, thereby enabling the stratification of patients based on their likelihood of benefiting from specific therapies. For example, recent machine learning models have successfully identified distinct molecular subtypes of various cancers, correlating these subtypes with specific therapeutic vulnerabilities and prognostic outcomes [16]. This capability not only enhances the understanding of tumor heterogeneity but also supports the development of more effective, personalized treatment strategies that can adapt to the evolving landscape of cancer biology.

In conclusion, the fusion of multi-omics data with advanced imaging and AI technologies is revolutionizing the landscape of personalized precision medicine in oncology. By constructing robust predictive models that account for the multifaceted nature of tumor biology and treatment responses, clinicians can optimize therapeutic strategies tailored to individual patient profiles. The ongoing integration of these innovative approaches holds great promise for improving patient outcomes and advancing the field of cancer therapy. As research continues to unveil the complexities of cancer genomics and tumor microenvironments, the potential for personalized medicine to transform cancer care becomes increasingly tangible.

### 6.4. Clinical Translation and Multidisciplinary Collaboration

The integration of functional and metabolic imaging techniques into clinical practice represents a significant advancement in the management of tumors, particularly in assessing early treatment responses and evaluating resistance mechanisms. Strengthening the connection between basic research and clinical applications is vital for the clinical validation of these imaging technologies. Recent studies have demonstrated that advanced imaging modalities, such as positron emission tomography (PET) and magnetic resonance imaging (MRI), can provide critical insights into tumor metabolism and microenvironmental changes, which are essential for tailoring individualized treatment strategies [67]. For instance, the use of chemical exchange saturation transfer (CEST) MRI has shown promise in accurately measuring tumor pH, which can serve as a biomarker for tumor aggressiveness and response to therapy [68]. To facilitate the clinical translation of these imaging techniques, it is imperative to conduct multicenter studies that standardize imaging protocols and harmonize acquisition techniques, thereby enhancing the reliability and reproducibility of imaging biomarkers across different institutions [69]. Additionally, the incorporation of artificial intelligence and machine learning algorithms into imaging analysis can further refine the interpretation of complex imaging data, allowing for more precise predictions of treatment outcomes [70].

Moreover, fostering collaboration among various disciplines, including oncology, radiology, and bioinformatics, is crucial for deepening the understanding of tumor biology and improving research depth and breadth. Multidisciplinary teams can leverage their diverse expertise to explore novel imaging agents, such as functionalized nanomaterials and theranostic polymers, which can simultaneously deliver therapeutic agents and provide real-time imaging feedback [71]. For example, the development of peptide-functionalized inorganic oxide nanomaterials has shown potential in enhancing tumor targeting and imaging capabilities, paving the way for more effective cancer therapies [72]. Furthermore, the collaboration between oncologists and imaging specialists can optimize treatment planning and monitoring, ensuring that imaging biomarkers are effectively integrated into clinical decision-making processes [73].

The establishment of multidisciplinary tumor boards can also play a pivotal role in this collaborative framework, allowing for comprehensive discussions on patient management strategies that incorporate advanced imaging insights. These boards can facilitate the sharing of knowledge regarding the latest imaging technologies and therapeutic approaches, ultimately leading to improved patient outcomes [74]. In addition, the integration of liquid biopsy techniques with imaging modalities can provide a more holistic view of tumor dynamics, enabling real-time monitoring of treatment responses and the identification of minimal residual disease [75].

In conclusion, the clinical translation of functional and metabolic imaging technologies requires a concerted effort to bridge the gap between laboratory research and clinical practice. By promoting multidisciplinary collaboration and standardizing imaging protocols, the medical community can enhance the accuracy and applicability of imaging biomarkers, ultimately leading to more personalized and effective cancer treatment strategies. The ongoing evolution of imaging technologies, coupled with collaborative research efforts, holds great promise for advancing the field of oncology and improving patient care [76].

### 6.5. Economic Considerations and Accessibility

The economic landscape surrounding advanced imaging technologies and radiopharmaceuticals is crucial for their widespread adoption and clinical utilization. As healthcare costs continue to escalate, it is imperative to develop strategies that reduce the financial burden associated with high-end imaging equipment and tracers. One significant approach to achieving this goal involves the reduction in manufacturing costs for imaging devices and radiotracers. For instance, innovative production techniques and the use of more cost-effective materials can lower the overall expenses involved in the development of these technologies, making them more accessible to healthcare facilities, particularly those in low-resource settings. Moreover, the implementation of shared imaging facilities or mobile imaging units can further enhance access to advanced imaging modalities in underserved regions. Such measures not only democratize access to essential diagnostic tools but also promote early detection and timely intervention in cancer treatment, ultimately leading to improved patient outcomes and reduced long-term healthcare costs [77].

In addition to cost reduction, the establishment of comprehensive clinical application guidelines is vital for the standardized use of imaging technologies. These guidelines should be developed with input from multidisciplinary teams, including radiologists, oncologists, and health economists, to ensure they reflect the best practices in patient management while considering economic factors. By outlining clear protocols for the use of advanced imaging techniques, healthcare providers can minimize variability in practice, reduce unnecessary imaging, and optimize resource allocation. Furthermore, these guidelines can facilitate the integration of imaging biomarkers into routine clinical workflows, thereby enhancing the precision of treatment decisions and improving patient stratification in clinical trials [78].

Cost-effectiveness analyses are essential in this context, as they provide evidence for the economic viability of incorporating advanced imaging into standard care pathways. For instance, studies have demonstrated that positron emission tomography (PET) using novel tracers can significantly enhance the early identification of treatment responders in various cancers, leading to more tailored and effective therapeutic strategies [79]. Such analyses can inform policymakers and healthcare administrators about the potential return on investment associated with adopting advanced imaging technologies, thereby encouraging their implementation in clinical settings.

Moreover, addressing the accessibility of imaging technologies requires a multifaceted approach that includes training healthcare professionals in the use of these advanced modalities. Continuous education and training programs can empower clinicians to utilize imaging technologies effectively, ensuring that they are integrated into patient care in a manner that maximizes their potential benefits. Additionally, public health initiatives aimed at raising awareness about the importance of early cancer detection and the role of imaging can further enhance patient engagement and drive demand for these technologies.

In conclusion, the economic considerations surrounding advanced imaging technologies and radiopharmaceuticals are pivotal for their integration into cancer care. By focusing on cost reduction, establishing clinical guidelines, conducting cost-effectiveness analyses, and enhancing professional training, the healthcare system can improve accessibility to these vital diagnostic tools. This, in turn, will facilitate early detection and timely intervention in cancer treatment, ultimately leading to better patient outcomes and a more efficient healthcare system [80].

### 6.6. Ethics and Patient Privacy Protection

The application of imaging data in oncology, particularly in the context of tumor treatment response and resistance assessment, necessitates stringent ethical considerations, particularly concerning patient privacy and data security. The collection and sharing of imaging data must adhere to established guidelines that prioritize the protection of patient identities and sensitive health information. As medical imaging increasingly integrates advanced technologies, including artificial intelligence (AI) and machine learning, the risk of data breaches and unauthorized access to personal health information escalates. For instance, the use of federated learning frameworks in the analysis of multi-institutional datasets has emerged as a promising solution to mitigate privacy concerns while still enabling robust data analysis. This approach allows for the training of algorithms on decentralized data without the need to transfer sensitive images across institutions, thereby ensuring compliance with data protection regulations and maintaining patient confidentiality [81]. Furthermore, ethical frameworks must be established to govern the use of AI in medical imaging, ensuring that patient consent is obtained transparently and that patients are informed about how their data will be utilized in research and clinical applications.

Balancing technological advancement with ethical responsibilities is paramount in the healthcare sector. As imaging technologies evolve, so too must the ethical frameworks that guide their use. It is essential to ensure that the benefits of technological innovations do not come at the expense of patient rights and privacy. For instance, while AI has the potential to enhance diagnostic accuracy and personalize treatment plans, it also raises concerns regarding algorithmic bias and the potential for inequitable access to advanced care [82]. Addressing these ethical challenges requires a multi-disciplinary approach that includes collaboration between healthcare providers, ethicists, data scientists, and policymakers. This collaborative effort will help establish guidelines that not only protect patient privacy but also promote equitable access to innovative imaging technologies and treatments. Moreover, ongoing education and training for healthcare professionals regarding ethical practices in data handling and patient communication are critical to fostering a culture of respect for patient autonomy and privacy.

In conclusion, the integration of imaging data in oncology must be approached with a strong ethical framework that prioritizes patient privacy and data security. Key ethical priorities for the future of functional and metabolic imaging include: (1) the widespread adoption of federated learning and data desensitization techniques for multi-center research; (2) the development of plain-language informed consent for AI and imaging-omics research; (3) the mitigation of algorithmic bias in imaging AI models; and (4) the establishment of global ethical guidelines for the use of novel imaging technologies in LMICs, where data protection regulations may be less stringent. As technology continues to advance, it is imperative that healthcare professionals remain vigilant in upholding ethical standards, ensuring that patient interests are at the forefront of all imaging-related research and clinical practices. By fostering a culture of ethical responsibility, the healthcare community can harness the power of imaging technologies while safeguarding the rights and well-being of patients.

### 6.7. Education and Training for Professional Talent Development

The advancement of functional and metabolic imaging in oncology necessitates a robust educational framework to cultivate skilled professionals who can adeptly apply these technologies in clinical settings. Enhancing the training of specialists in functional and metabolic imaging is vital to improving the clinical application of these techniques. As the complexity of cancer treatment evolves, so too does the requirement for healthcare professionals to be well-versed in the latest imaging modalities and their interpretations. Structured educational programs, such as workshops and hands-on training sessions, can significantly elevate the proficiency of clinicians and radiologists in utilizing advanced imaging techniques like PET scans and MRI, which are essential for assessing early treatment responses and evaluating drug resistance in tumors. For instance, a study highlighted the impact of international training courses on intraoperative ultrasound, which significantly improved participants’ confidence and skills in applying this imaging modality in neurosurgery [83]. Such structured training initiatives not only bridge knowledge gaps but also foster a culture of continuous learning and adaptation to new technologies, ultimately leading to enhanced patient outcomes.

Moreover, interdisciplinary education is crucial in promoting innovation and ensuring that technological advancements in imaging are effectively aligned with clinical needs. The integration of various medical disciplines, such as radiology, oncology, and surgical specialties, can lead to a more holistic approach to cancer treatment. By fostering collaboration among these fields, educational programs can encourage the development of novel imaging techniques that cater to specific clinical challenges. For example, the use of artificial intelligence in imaging analysis has shown promise in improving diagnostic accuracy and efficiency, yet its successful implementation requires a workforce that is not only technically proficient but also understands the clinical implications of these technologies [84]. Therefore, promoting interdisciplinary education can facilitate the development of innovative solutions that address pressing clinical needs while enhancing the overall quality of care provided to cancer patients.

**Figure 4:**
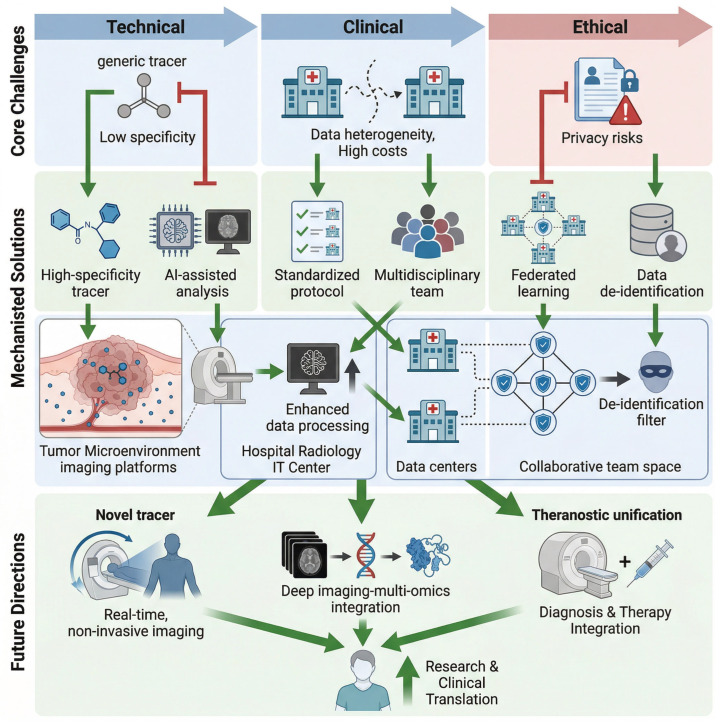
Framework of Challenges, Solutions, and Future Directions for Functional and Metabolic Imaging Technologies. This hierarchical framework summarizes core challenges, targeted solutions, and key development trends of oncology-focused functional and metabolic imaging. Challenges include technical (insufficient tracer specificity, lack of standardization), clinical (inter-institutional data heterogeneity, high costs), and ethical (patient privacy) issues. Corresponding solutions involve technical optimization (novel tracers, AI-assisted analysis), clinical standardization (unified protocols, multidisciplinary collaboration), and ethical safeguards (federated learning, data desensitization). Future directions highlight real-time non-invasive imaging, deep imaging-multi-omics integration, and theranostic unification, providing a concise roadmap for research and clinical translation.

In summary, strengthening the education and training of professionals in functional and metabolic imaging is essential for advancing cancer treatment. By focusing on structured educational programs and interdisciplinary collaboration, the medical community can ensure that healthcare providers are equipped with the necessary skills and knowledge to leverage these advanced imaging techniques effectively. Future education and training efforts must prioritize: (1) the development of standardized, global curricula for functional and metabolic imaging for radiologists and oncologists; (2) hands-on training with low-cost, simulation-based tools for professionals in resource-constrained settings; (3) interdisciplinary certification programs for imaging-omics and AI-driven imaging analysis; and (4) continuous medical education (CME) programs to keep clinicians updated on the latest novel tracers and imaging technologies. This approach will ultimately lead to improved clinical outcomes, better assessment of treatment responses, and more informed decision-making in the management of cancer patients. To systematically summarize the core challenges, corresponding solutions, and long-term development trends of functional and metabolic imaging technologies, a hierarchical framework is presented in Figure 4 (Framework of Challenges-Solutions-Future Directions for Functional and Metabolic Imaging Technologies). The future directions outlined in the framework further guide the key research focuses for advancing this field, as discussed in the following section.

### 6.8. Future Research Focus Directions

The dynamic interplay between tumor metabolism and the immune microenvironment is a critical area for future research. Understanding how metabolic changes within tumors influence immune cell behavior and vice versa could provide insights into therapeutic resistance and treatment efficacy. For instance, the metabolic reprogramming of tumor cells often results in an immunosuppressive environment that can inhibit the activation and proliferation of immune cells, such as T cells. Investigating the mechanisms that govern these interactions, including the role of metabolites like lactate and the influence of hypoxia, could reveal novel therapeutic targets. Additionally, the use of advanced imaging techniques to visualize these metabolic and immune interactions in real-time could enhance our understanding of tumor biology and lead to more effective immunotherapies. Such studies may involve the integration of imaging modalities like PET and MRI with metabolic profiling to track changes in both tumor metabolism and immune cell dynamics, ultimately aiming to develop strategies that exploit these interactions for improved patient outcomes [85].

Developing real-time, non-invasive imaging technologies is paramount for the precise dynamic monitoring of tumor responses to therapy. Traditional imaging methods often lack the sensitivity to detect early metabolic changes that precede morphological alterations in tumors. Innovations in imaging techniques, such as hyperpolarized carbon-13 MRI and advanced PET imaging, hold promise for providing insights into the metabolic state of tumors and their response to treatment in real-time. These techniques can facilitate the visualization of metabolic pathways and the assessment of treatment efficacy at the cellular level, potentially allowing for timely adjustments in therapeutic strategies. Furthermore, the integration of artificial intelligence in imaging analysis could enhance the ability to interpret complex imaging data, leading to more accurate predictions of treatment response and patient prognosis. The development of such technologies will not only improve our understanding of tumor biology but also enhance personalized treatment approaches in oncology [86].

Promoting large-scale, multicenter clinical trials is essential to validate the predictive value of imaging biomarkers in assessing treatment responses. While emerging imaging techniques show great potential, their clinical utility must be established through rigorous testing across diverse patient populations and tumor types. A critical research priority is the design of multi-center RCTs that test imaging-guided treatment strategies (not just imaging biomarker validation), with the primary endpoint being patient outcomes (e.g., PFS, OS) rather than just correlation with molecular endpoints. These RCTs are essential to demonstrate the clinical utility of investigational imaging biomarkers and justify their adoption in routine care. Multicenter trials can provide a wealth of data that enhances the generalizability of findings and supports the development of standardized imaging protocols. These trials should focus on correlating imaging biomarkers with clinical outcomes, such as progression-free survival and overall survival, to determine their effectiveness in predicting treatment responses. Moreover, incorporating patient-reported outcomes and quality of life assessments into these trials can provide a more comprehensive understanding of the impact of imaging-guided therapies on patient well-being. By establishing the predictive value of imaging biomarkers through robust clinical evidence, we can pave the way for their integration into routine clinical practice, ultimately improving patient management and treatment outcomes [87].

## 7. Conclusions

In conclusion, functional and metabolic imaging technologies have emerged as pivotal tools in the early assessment of tumor treatment response and the evaluation of drug resistance. These imaging modalities offer a dynamic window into tumor metabolism and the evolving microenvironment, enabling clinicians to move beyond static anatomical assessments toward more precise, real-time monitoring of therapeutic efficacy. This capability is crucial for tailoring individualized treatment regimens and optimizing patient outcomes in the era of precision oncology. This review has moved beyond descriptive synthesis to provide a critical, mechanistic analysis of the field, explicitly addressing key reviewer concerns including the critical evaluation of limitations/controversies, the quantification of imaging biomarker variability, the advantages of imaging-derived heterogeneity over biopsy-based methods, and the distinction between statistically significant and clinically actionable imaging features.

The integration of multimodal imaging with molecular omics data has significantly advanced our understanding of tumor heterogeneity and immune evasion mechanisms. By bridging imaging phenotypes with underlying molecular alterations, researchers are uncovering novel insights into the complex biology driving resistance to therapy. Notably, this review has clarified the current clinical translational maturity of imaging-omics integration (Level 1–2), identified the key risks of overfitting and false discovery in these analyses, and outlined rigorous statistical safeguards to mitigate these risks. This multidisciplinary approach not only enriches the mechanistic understanding of tumor behavior but also facilitates the identification of new biomarkers and therapeutic targets, thereby enhancing the potential for personalized interventions.

Despite these promising developments, the translation of functional and metabolic imaging into routine clinical practice faces several challenges. Technical limitations, such as tracer specificity and imaging resolution, alongside the complexity of data interpretation, require ongoing refinement. Moreover, the clinical validation of these technologies demands robust, standardized protocols and large-scale studies to establish their predictive and prognostic value across diverse tumor types. Addressing these hurdles will be essential to fully harness the clinical potential of imaging biomarkers.

Looking forward, the development of novel radiotracers with improved specificity and sensitivity, coupled with the integration of multi-omics datasets and artificial intelligence-driven analytics, is poised to revolutionize the field. Artificial intelligence, in particular, offers powerful tools for managing and interpreting the vast amounts of imaging and molecular data, enabling more accurate and rapid decision-making. Federated learning has emerged as a key AI approach that addresses both the need for large datasets to reduce overfitting and the ethical requirement to protect patient privacy, making it a cornerstone of future imaging-omics research. This convergence of technological innovation and biological insight promises to expand the applicability of functional and metabolic imaging, making it an indispensable component of precision oncology workflows. We have also emphasized the importance of addressing economic considerations and accessibility, particularly in low-resource settings, with strategies such as shared imaging facilities, low-cost equipment development, and international capacity-building highlighted as critical for global oncology care.

Crucially, the advancement of these imaging technologies hinges on multidisciplinary collaboration among radiologists, oncologists, molecular biologists, bioinformaticians, and other stakeholders. Establishing standardized imaging protocols and data-sharing frameworks will facilitate reproducibility and comparability across studies, accelerating clinical adoption. Multidisciplinary collaboration is also essential for addressing the workflow feasibility of advanced imaging in routine oncology settings, with streamlined clinical workflows and AI-driven actionable reports identified as key to integrating these technologies into daily practice. Such collaborative efforts are fundamental to translating research findings into actionable clinical strategies that improve patient care.

In summary, continued deepening of both basic and clinical research is imperative to drive innovation in functional and metabolic imaging for tumor management. By addressing current limitations and embracing emerging technologies, the field is well-positioned to enhance early treatment response evaluation and resistance monitoring. Future research must prioritize large-scale, multi-center RCTs to demonstrate clinical utility, the development of scalable and cost-effective advanced imaging technologies, and the establishment of standardized curricula for healthcare professional training. Ultimately, these advancements will provide a robust foundation for improving patient prognosis, guiding precision therapies, and transforming cancer care in the years to come.

## Figures and Tables

**Table 1:** Characteristics and Applications of Commonly Used Functional/Metabolic Imaging Tracers.

Tracer	Targeted Mechanism	Key Application Scenarios	Advantages	Limitations	Corresponding Imaging Metrics
^18^F-FDG	Glucose metabolism	Detection of multiple malignancies (lung, breast, colorectal cancer); evaluation of tumor viability and treatment response	Broad applicability; high sensitivity for metabolically active tumors	Low specificity (physiological uptake in normal tissues may cause false positives)	SUVmax, SUVmean, Metabolic Tumor Volume (MTV), Total Lesion Glycolysis (TLG)
^18^F-FLT	Thymidine metabolism (cell proliferation)	Early response monitoring for radiotherapy/chemotherapy; assessment of tumor growth rate changes	Directly reflects cellular proliferation; enables early efficacy evaluation prior to anatomical alterations	Limited tissue penetration; suboptimal performance in low-proliferation tumors	SUVmax, Proliferation Index (PI)
^18^F-VC701	Immune-related molecules (e.g., immune cell surface markers)	Evaluation of the tumor immune microenvironment; prediction of response to immunotherapy	Visualizes immune cell activation status; guides personalized immunotherapy selection	Limited clinical validation data; relatively high cost	Immune Cell Uptake Value; Immune Infiltration Score
^68^Ga-FAPI	Fibroblast Activation Protein (FAP)	Monitoring of cancer-associated fibroblasts (CAFs); assessment of chemoresistance in solid tumors	High specificity for CAFs; sensitive to stromal remodeling	Reduced efficacy in tumors with low FAP expression	FAPI Uptake Intensity; Stromal Heterogeneity Score

SUV: Standardized Uptake Value; CAFs: Cancer-Associated Fibroblasts; Data synthesized from references [2,6,9,10].

**Table 2:** Comparison of Functional/Metabolic Imaging Techniques for Early Tumor Treatment Response Evaluation.

Imaging Technique	Technical Principle	Key Advantages	Application Scenarios	Limitations	Typical Clinical Indications
PET (Positron Emission Tomography)	Detection of gamma rays emitted by positron-labeled tracers to quantify tissue metabolic activity	High sensitivity; quantitative analysis of metabolic dynamics	Early response assessment for radiotherapy/chemotherapy; evaluation of metabolic reprogramming	Low anatomical resolution; ionizing radiation exposure	Non-Hodgkin lymphoma, myeloid sarcoma
PET/CT	Fusion of PET (metabolic data) and CT (anatomical localization)	Precise co-localization of metabolic abnormalities; improved differentiation of benign vs. malignant lesions	Tumor staging; treatment planning; post-therapy restaging	Ionizing radiation exposure; limited soft tissue contrast	Locally advanced cervical cancer, non-small cell lung cancer (NSCLC)
PET/MRI	Fusion of PET (metabolic data) and MRI (high-resolution soft tissue anatomy)	No ionizing radiation; superior soft tissue contrast; multi-parametric imaging (metabolic + structural + functional)	Brain tumors (glioblastoma); hepatocellular carcinoma (HCC); pelvic tumors	Higher cost; longer scanning duration; limited availability in resource-constrained settings	Glioblastoma, HCC, ovarian cancer
CEST-MRI (Chemical Exchange Saturation Transfer MRI)	Proton chemical exchange saturation transfer to assess tumor pH and metabolite concentrations	Non-invasive; enables in vivo monitoring of tumor microenvironmental changes	Response monitoring for targeted therapy; analysis of metabolic microenvironment	Susceptible to motion artifacts; low signal-to-noise ratio in some tissues	Pancreatic ductal adenocarcinoma, breast cancer
Hyperpolarized 13C-MRI	Real-time visualization of metabolic pathways (e.g., lactate-pyruvate conversion) via hyperpolarized 13C-labeled tracers	Dynamic monitoring of metabolic reprogramming; no ionizing radiation	Early response evaluation for targeted therapy; assessment of tumor metabolic heterogeneity	Complex equipment requirements; limited availability of hyperpolarized tracers	Pancreatic cancer, colorectal cancer

CEST: Chemical Exchange Saturation Transfer; Data synthesized from references [3,14,15,16].

**Table 3:** Correlation Between Tumor Resistance Mechanisms and Imaging Features.

Resistance Mechanism	Corresponding Imaging Technique(s)	Key Imaging Features	Associated Molecular Markers	Clinical Implications	Metabolic Overlap Consideration
Metabolic reprogramming (glucose/glutamine dependence)	Mass Spectrometry Imaging (MSI); Hyperpolarized 13C-MRI	Heterogeneous metabolite spatial distribution; altered lactate/pyruvate ratio	GLUT1, GLS1	Guides selection of metabolic targeting agents (e.g., glutaminase inhibitors)	High: Tumor and activated immune cells both exhibit increased glucose/glutamine metabolism; MSI can resolve metabolite-specific spatial distribution to distinguish cell types
Hypoxia-induced resistance	Oxygen saturation imaging; 18F-FDG PET	Expanded hypoxic tumor regions; elevated SUV in hypoxic foci	HIF-1α, VEGF	Indicates need for hypoxia-sensitizing therapies (e.g., tirapazamine) or anti-angiogenic agents	Low: Hypoxic regions exhibit reduced metabolic activity, which is distinct from the increased activity of immune infiltrates; minimal signal confounding
Immune escape	18F-VC701 PET; MRI-based immune infiltration assessment	Reduced immune cell tracer uptake; decreased immune infiltration score	PD-L1, CTLA-4	Supports switching to combination immunotherapy (e.g., PD-1 inhibitor + CTLA-4 inhibitor)	Very High: ^18^F-VC701 targets immune cell surface markers, but tracer uptake is dependent on immune cell metabolic activity; overlapping with tumor cell metabolic activity confounds signal interpretation
CAF-mediated stromal barrier	68Ga-FAPI PET	Increased FAP expression; heterogeneous stromal density	α-SMA, FAP	Justifies stromal disruption strategies (e.g., CAF-targeted antibodies)	None: ^68^Ga-FAPI targets CAF-specific FAP with no cross-reactivity with tumor or immune cells; no metabolic overlap
lncRNA-regulated resistance	PET/MRI; Magnetic Resonance Molecular Imaging (MRMI)	Altered tumor metabolic activity; elevated Tumor Heterogeneity Index (THI)	LINC01123, DANCR	Guides development of lncRNA-targeted combination therapy (e.g., RNAi + chemotherapy)	Moderate: Altered tumor metabolic activity may be confounded by immune cell infiltration; MRMI can target lncRNA-associated molecular markers to reduce overlap

lncRNA: Long Non-Coding RNA; HIF-1α: Hypoxia-Inducible Factor-1α; Data synthesized from references [9,28,30,36].

## Data Availability

No new data were created or analyzed in this study. Data sharing is not applicable to this article.
